# Vitamin D and Its Analogues: From Differences in Molecular Mechanisms to Potential Benefits of Adapted Use in the Treatment of Alzheimer’s Disease

**DOI:** 10.3390/nu15071684

**Published:** 2023-03-30

**Authors:** Andrea Thiel, Carina Hermanns, Anna Andrea Lauer, Jörg Reichrath, Tobias Erhardt, Tobias Hartmann, Marcus Otto Walter Grimm, Heike Sabine Grimm

**Affiliations:** 1 Experimental Neurology, Saarland University, 66424 Homburg, Germany; 2 Nutrition Therapy and Counseling, Campus Rheinland, SRH University of Applied Health Sciences, 51377 Leverkusen, Germany; 3 Department of Dermatology, Saarland University Hospital, 66421 Homburg, Germany; 4 Physical Therapy, Campus Karlsruhe, SRH University of Applied Health Sciences, 76185 Karlsruhe, Germany; 5 Deutsches Institut für DemenzPrävention, Saarland University, 66424 Homburg, Germany

**Keywords:** vitamin D, vitamin D derivatives, vitamin D analogues, Alzheimer’s disease, vitamin D receptor, vitamin D binding protein, 1,25-dihydroxyvitamin D_3_, 1,25(OH)_2_D_3_

## Abstract

Lifestyle habits and insufficient sunlight exposure lead to a high prevalence of vitamin D hypovitaminosis, especially in the elderly. Recent studies suggest that in central Europe more than 50% of people over 60 years are not sufficiently supplied with vitamin D. Since vitamin D hypovitaminosis is associated with many diseases, such as Alzheimer’s disease (AD), vitamin D supplementation seems to be particularly useful for this vulnerable age population. Importantly, in addition to vitamin D, several analogues are known and used for different medical purposes. These vitamin D analogues differ not only in their pharmacokinetics and binding affinity to the vitamin D receptor, but also in their potential side effects. Here, we discuss these aspects, especially those of the commonly used vitamin D analogues alfacalcidol, paricalcitol, doxercalciferol, tacalcitol, calcipotriol, and eldecalcitol. In addition to their pleiotropic effects on mechanisms relevant to AD, potential effects of vitamin D analogues on comorbidities common in the context of geriatric diseases are summarized. AD is defined as a complex neurodegenerative disease of the central nervous system and is commonly represented in the elderly population. It is usually caused by extracellular accumulation of amyloidogenic plaques, consisting of amyloid (Aβ) peptides. Furthermore, the formation of intracellular neurofibrillary tangles involving hyperphosphorylated tau proteins contributes to the pathology of AD. In conclusion, this review emphasizes the importance of an adequate vitamin D supply and discusses the specifics of administering various vitamin D analogues compared with vitamin D in geriatric patients, especially those suffering from AD.

## 1. Clinical Relevance of Vitamin D and Its Analogues

**Figure 1:**
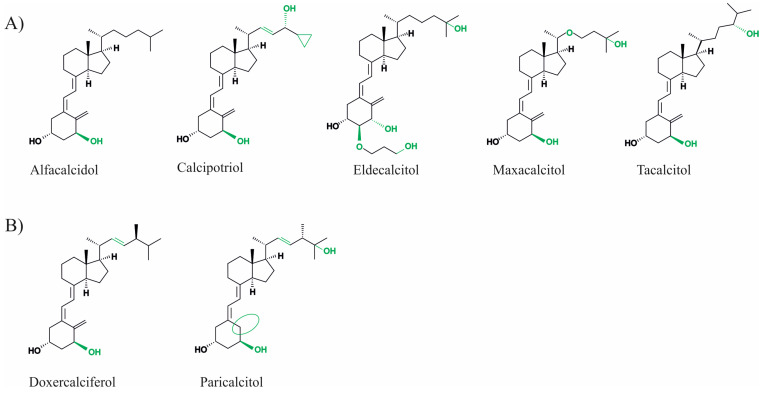
Chemical structure of vitamin D_3_ derivatives (**A**) and vitamin D_2_ analogues (**B**). Structural changes between the analogues are highlighted in green or with green circles.

Vitamin D is a lipophilic secosteroid, which plays an important role in the regulation of the processes of phosphorus–calcium metabolism, the immune system, cell proliferation and differentiation, and other crucial functions within the body. Furthermore, an adequate supply of vitamin D is important for bone health. Vitamin D status, which can be measured by the serum concentration of 25-OH vitamin D_3_, should be above 50 nmol/L, according to the International Institute of Medicine. 25-OH vitamin D_3_ serum concentrations between 30 and 50 nmol/L are considered to be suboptimal and at concentrations below 30 nmol/L vitamin D deficiency is present [1]. Vitamin D deficiency has a high prevalence worldwide, for example, 40.4% of the European population has inadequate vitamin D levels (<50 nmol/L). Levels below 30 nmol/L have been reported in 13% of the European and 5.9% of the American populations [2,3]. These deficits can be attributed to an inadequate supply of vitamin D via the diet, sun exposure, or supplementation, which may result from poor lifestyle habits. Physical inactivity, a home bound lifestyle, or an unbalanced diet, particularly in old age, can contribute to inadequate vitamin D serum concentrations [4]. Vitamin D deficiency is associated with various chronic diseases, such as diabetes or arterial hypertension, and leads to bone mineralization disorders, including rickets in children or osteoporosis and osteomalacia in adults (reviewed in detail in [5]). Furthermore, associations with neurological diseases such as Alzheimer’s disease (AD), Parkinson’s disease, and multiple sclerosis, renal insufficiency, autoimmune diseases, and even cancer have been discussed [6,7]. Therefore, several studies investigated the preventive and therapeutic benefits of vitamin D supplementation. In addition to cholecalciferol (vitamin D_3_), ergocalciferol (vitamin D_2_), and calcidiol (25-OH vitamin D_3_), which are still the most prescribed vitamin D preparations, several synthetic vitamin D analogues have also been developed. These drugs aim to act in special cases more efficiently, more selectively and have fewer side effects due to structural modifications of vitamin D [8,9]. In this process, the basic structure of vitamin D, which consists of an A-ring, a seco-B-ring, a CD-ring, and a side chain, is modified at different points. For example, 19-nor analogues, such as paricalcitol, have two hydrogen atoms in the A-ring at the 19-carbon atom instead of the exocyclic methyl group. Maxacalcitol has an additional oxygen atom in the side chain. Vitamin D_2_ differs from D_3_ by a double bond in the side chain and an additional methyl group. They are both analogues derived from vitamin D_3_ and derivatives based on the basic structure of vitamin D_2_ (see Figure 1).

More than 3000 vitamin D analogues have been developed so far and some have received regulatory approval and are currently used worldwide in various medical fields, including dermatology, nephrology, and endocrinology. For example, the 1α-hydroxylated vitamin D analogues alfacalcidol, paricalcitol, and doxercalciferol—also maxacalcitol and falecalcitriol in Japan—are used in the therapy of secondary hyperparathyroidism. Calcitriol, tacalcitol, and calcipotriol—and maxacalcitol in Japan—are prescribed for the inflammatory skin disease psoriasis vulgaris. Additionally, calcitriol, alfacalcidol, and eldecalcitol are utilized for the prevention and therapy of osteoporosis [10,11,12].

In diseases such as osteoporosis or secondary hyperparathyroidism, vitamin D is used by intervening in bone metabolism by regulating calcium and phosphate homeostasis. Additionally, the antiproliferative, cell differentiation-promoting, and immunosuppressive effects of individual vitamin D analogues are utilized in the treatment of psoriasis vulgaris [10,13]. Table 1 gives an overview of the main indications of vitamin D analogues and their mechanism of action. 

Although vitamin D analogues are commonly used for the treatment of various diseases, including osteoporosis and chronic kidney disease (CKD), the use of vitamin D analogues is associated with potential risks, such as hypercalcemia, hyperphosphatemia, and hypercalciuria. Several studies have reported an increased risk of these complications with the use of vitamin D analogues compared to cholecalciferol and ergocalciferol. For instance, a randomized controlled trial by Sprague et al. reported a significantly higher risk of hypercalcemia and hypercalciuria in patients receiving vitamin D analogues compared to those receiving cholecalciferol or placebo [14]. Similarly, a systematic review and meta-analysis by Bolland et al. reported a higher risk of hypercalcemia and hyperphosphatemia with vitamin D analogue use in patients with CKD [15]. These findings suggest that the use of vitamin D analogues should be carefully considered and monitored, especially in patients at high risk of these complications. The limitations and risks of vitamin D analogue use in clinical practice should be taken into account when making treatment decisions, and alternative strategies, such as dose titration and close monitoring, should be considered to minimize the risk of these complications.

**Table 1:** Summary of the various vitamin D analogues approved as drugs, their indication in relation to different illnesses, and the mechanism of action within the body.

Indication	Approved Drug	Mechanism of Action
rickets/osteomalacia	cholecalciferol, ergo-calciferol, calcidiol (25-hydroxycholecalciferol), calcitriol	increased reabsorption of calcium and phosphate in the intestine and kidney increase in bone mineral density [16]
osteoporosis, renal osteopathy	cholecalciferol, ergo-calciferol, calcitriol, alfacalcidol, eldecalcitol (Japan)	
psoriasis	calcitriol, calcipotriol, tacalcitol, maxacalcitol (Japan)	inhibition of proliferation of epidermal keratinocytes and T-lymphocytes inhibition of chemokines that trigger psoriasis [12,13]
hypoparathyroidism	calcitriol, alfacalcidol	compensation of the vitamin D deficit, which resulted from the lack of vitamin D synthesis due to parathyroid hormone deficiency [17,18]
chronic renal insufficiency, hyperparathyroidism	calcitriol, doxercalciferol, alfacalcidol, paricalcitol, maxacalcitol (Japan), fale-calcitriol (Japan)	reduction of parathyroid hormone release [8,19,20]

**Figure 2:**
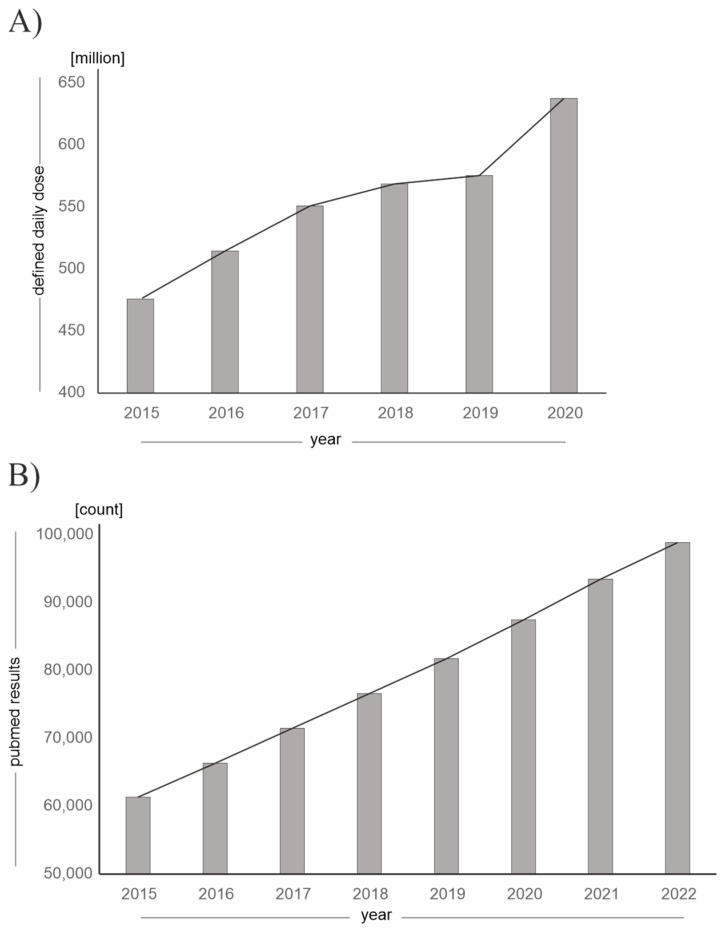
Increasing clinical and environmental relevance of vitamin D and its analogues. (**A**) Defined daily dose (DDD) of vitamin D and vitamin D analogues prescribed in Germany, which has been increasing since 2015 until 2020. (Source: annual “Arzneiverordnungs-Report” in the publishing company “Springer”, data given in million DDD). (**B**) Increasing number of publications on PubMed for the search term “vitamin D”, “vitamin D analogues” or “vitamin D derivatives” as of 30 December 2022.

The fact that vitamin D and its analogues are becoming increasingly clinically and environmentally relevant is reflected in rising prescription rates and an increase in publications on the subject, as shown in Figure 2.

The increasing prescription rates can additionally be attributed to the concurrent SARS-CoV-2 pandemic, as the benefits of vitamin D supplementation with regard to prophylaxis and therapy of SARS-CoV-2 infection were discussed [21,22] and parts of the population took vitamin D supplements on their own initiative. In addition, the incidence of various diseases for which vitamin D analogues are approved is constantly rising [23]. For example, the number of patients with advanced renal insufficiency is increasing: by 2040, an increase of 20–23% in patients requiring dialysis is expected in Germany. Furthermore, vitamin D, as well as vitamin D analogues, are an important pillar in the therapy of postoperative hypoparathyroidism, the incidence of which is rising due to the increasing number of thyroid operations [24,25].

This review aims to address the necessity for an adequate vitamin D supply, especially in vulnerable populations such as patients with AD and other comorbidities. It emphasizes the probable beneficial application possibilities for some of the approved vitamin D analogues. Furthermore, the individual benefits, the mechanisms of action, side effects and their implication for AD are discussed extensively in the following sections.

## 2. Metabolic Steps of Synthesis of Vitamin D and Its Analogues

**Figure 3:**
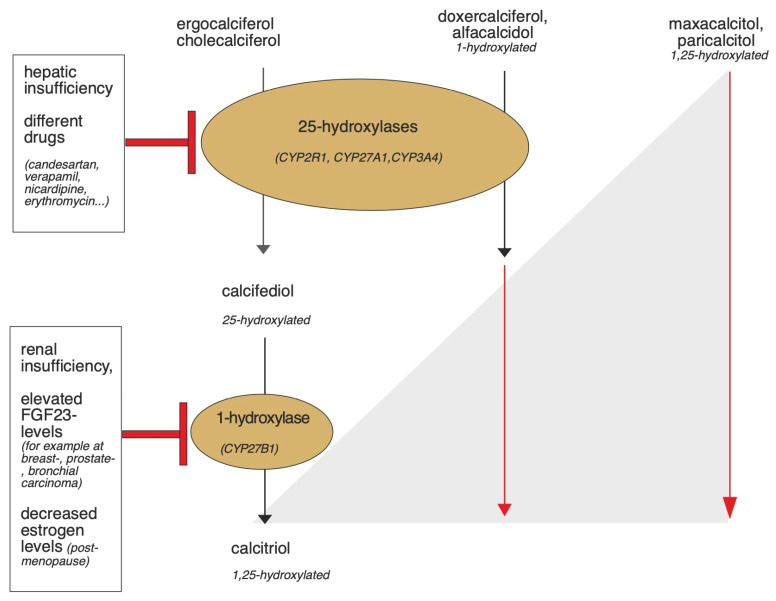
Graphical summary of the activation of vitamin D and vitamin D analogues by the 1- and 25-hydroxylases and potential triggers that may lead to enzyme dysfunction. In hepatic insufficiency or due to the influence of certain drugs, the function of 25-hydroxylases can be impaired [28,29,30]. Renal insufficiency, elevated FGF23 levels, and decreased estrogen levels may affect the functionality of 1-hydroxylase [31,32,33,34]. While cholecalciferol and ergocalciferol are activated by the 1- and 25-hydroxylases, the activation of some synthetic vitamin D analogues occurs independently of renal and extrarenal 1-hydroxylase, because they are already hydroxylated at the first carbon atom. Calcitriol, doxercalciferol, and alfacalcidol need only be activated by 25-hydroxylase. Maxacalcitol and paricalcitol bypass both 1 and 25-hydroxylation since they are already hydroxylated at the 1st and 25th carbon atom [35].

Physiologically, vitamin D requirements are met in the form of ergocalciferol or cholecalciferol via the diet or, with sufficient sunlight, 80–90% is achieved by the body’s own synthesis. De novo synthesis begins in the liver by dehydration of cholesterol to 7-dehydrocholesterol (7-DHC). Bound to the vitamin D binding protein (DBP), 7-DHC enters the skin via the bloodstream, where cholecalciferol is formed by ultraviolet B radiation with a wavelength of 290–315 nm [26]. Subsequently, both exogenously ingested and cutaneous formed cholecalciferol are transported to the liver bound to DBP and hydroxylated by 25-hydroxylases, which belong to the superfamily of cytochrome P450 enzymes (CYP, mainly CYP2R1/CYP27A1) to calcidiol. Calcidiol is converted to calcitriol (1,25(OH) vitamin D_3_), the most active metabolite of vitamin D, by 1α-hydroxylase (CYP27B1), particularly in the kidney, but also in other target tissues [27]. The vitamin D analogues already carry different hydroxylation modifications, so they can bypass some of these steps. Figure 3 provides an overview, and the individual steps are described in more detail below.

### 2.1. 25-Hydroxylases and Vitamin D Analogues

The 25-hydroxylation that converts cholecalciferol and ergocalciferol to calcidiol, can be catalyzed by various enzymes and occurs mainly in the liver. Patients with liver failure can show decreased expression of 25-hydroxylases. However, this synthesis step can also take place in extrahepatic tissues, including the bone marrow, brain, or adipose tissue. Under physiological conditions, CYP2R1 appears to be the most relevant 25-hydroxylase [16,36]. Particularly in the 25-hydroxylation of vitamin D analogues such as doxercalciferol or alfacalcidol, the 25-hydroxylase CYP27A1 also has an important pharmacological role. CYP27A1 are known to be inhibited by concurrent treatment with antihypertensive drugs or drugs against Human Immunodeficiency Virus (HIV) or cancer, as shown in Table 2. In addition to CYP2R1 and CYP27A1, CYP3A4 has also been shown to potentially act as a 25-hydroxylase. Here, CYP3A4 hydroxylates vitamin D_2_ substrates more efficiently than vitamin D_3_ substrates. In addition to the ability to 25-hydroxylate, 24-hydroxylation and thus catabolism of vitamin D analogues appears to be the major role of CYP3A4. CYP3A4 metabolizes up to 50% of drugs and is therefore liable to numerous interactions [37]. However, the extent to which concomitant use of these drugs affects the levels and clinical effects of vitamin D analogues remains to be demonstrated in clinical trials.

If 25-hydroxylase capacity is inhibited by concomitant use of inhibitory drugs or impaired liver function, the use of 25-hydroxylated supplements may be beneficial. Calcidiol, calcitriol, or 25-hydroxylated vitamin D analogues such as the vitamin D_3_ derivative maxacalcitol or the vitamin D_2_ derivative paricalcitol could be used for this purpose.

### 2.2. 1α-Hydroxylase and Vitamin D Analogues

Furthermore, the use of 1α-hydroxylated vitamin D analogues can bypass renal or extrarenal 1α-hydroxylase (CYP27B1), which converts calcidiol to its active form calcitriol. 1α-hydroxylase belongs also to the superfamily of cytochrome P450 enzymes and is controlled mainly by calcium, parathyroid hormone, and calcitriol in renal and extrarenal tissues mainly by cytokines. However, different pathomechanisms and drug interactions may also affect the expression and activity of 1α-hydroxylase, thereby inhibiting calcitriol synthesis [31,32,33,34].

Renal 1α-hydroxylase decreases in renal insufficiency. Approximately 2 million people in Germany suffer from renal insufficiency. According to the German Health Interview and Examination Survey for Adults (DEGS), which is part of The Robert Koch Institute’s German health-monitoring program, limitations in kidney function occur in one out of eight people aged from 70 to 79 [40]. If untreated, this can lead to disturbances in mineral and bone balance and contribute to the development of secondary hyperparathyroidism. To prevent this, 1α-hydroxylated vitamin D analogues, such as alfacalcidol, paricalcitol, or doxercalciferol, are approved for the substitution of vitamin D deficiency in advanced renal failure and can increase calcitriol levels independently of renal function [16,41]. In addition, vitamin D analogues may contribute to the preservation of renal function in renal failure. For example, in a study of patients with diabetic nephropathy, daily administration of 2 µg paricalcitol resulted in a significant improvement in albuminuria [42]. A randomized, blinded trial in vitamin D deficient patients with advanced renal failure showed that three months of supplementation with 1 µg doxercalciferol daily reduced initially elevated parathyroid hormone levels by 27% [43]. 

It has also been shown that elevated uric acid levels, as seen in gouty disease, decrease protein levels and the expression of 1α-hydroxylase [41,44]. Hyperglycemia, which occurs in diabetes mellitus, decreased 1α-hydroxylase expression in cell models and low parathyroid hormone levels in the context of hypoparathyroidism also reduced 1α-hydroxylase [16,45]. Mutations in the 1α-hydroxylase gene lead to autosomal recessive inherited pseudo vitamin D deficiency rickets type 1 [46]. Furthermore, renal and extrarenal 1α-hydroxylase is also regulated by fibroblast growth factor 23 (FGF23). Mice injected with FGF23 exhibited a dose-dependent reduction in renal 1α-hydroxylase mRNA levels, and 1α-hydroxylase expression in monocytes from human serum samples was also decreased after incubation with FGF23 [47,48,49,50,51]. Diseases leading to increased FGF23 concentrations might therefore be associated with decreased 1α-hydroxylase levels. Different tumor diseases, such as breast carcinoma, prostate carcinoma, bronchial carcinoma, or urothelial carcinoma are able to produce FGF23 [52]. Therefore, in these diseases, the use of 1α-hydroxylated vitamin D analogues, to circumvent the 1α-hydroxylase deficiency, may be beneficial; however, further clinical studies are needed to prove the advantages of these analogues. 

1α-hydroxylase also appears to function more inefficiently with age. In one study, calcitriol levels decreased with age, whereas calcidiol levels remained constant [53]. In addition, another study showed that young rats metabolized 20.8% of an orally administered calcidiol preparation to calcitriol within 24 h, whereas older rats converted only 2.1% in the same time [54]. Moreover, 1α-hydroxylase is affected by estrogen levels and therefore might contribute to decreased postmenopausal renal synthesis of calcitriol [55,56,57].

### 2.3. Vitamin D Binding Protein and Vitamin D Analogues

In addition to bypassing anabolic enzymes of vitamin D synthesis, the vitamin D analogues have further important pharmacological features. For example, modification of the side chain of the backbone of vitamin D alters its binding properties. This leads to a reduced affinity for DBP, which has several consequences. For example, the rate of cellular uptake is affected because the lower affinity for DBP results in a larger gradient into the cell with the same affinity for the intracellular VDR. This results in faster uptake of the vitamin D analogues into the target cell and a faster increase in intracellular drug levels. Moreover, the degradation of vitamin D analogues is also affected, as unbound metabolites are degraded more rapidly [58]. Some vitamin D analogues use alternative transport mechanisms in the blood due to their reduced affinity to DBP. Maxacalcitol, for example, is mainly transported by chylomicrons, which is due to the fact that the binding affinity of maxacalcitol to DBP in rat plasma is 600 times lower compared to calcitriol [59]. This alternative transport of maxacalcitol might be useful in diseases associated with decreased DBP concentrations, such as malnutrition, hepatic insufficiency, or increased DBP excretion in the context of proteinuria.

## 3. Side Effects of Vitamin D Analogues

The altered pharmacokinetics and pharmacodynamics of vitamin D analogues not only change their efficacy, but also their side effect profile. Some vitamin D analogues act more selectively and in a more tissue-specific manner [9]. For example, some vitamin D analogues act less effectively in the intestine, which means that calcium absorption is less affected, and these vitamin D analogues are less likely to cause hypercalcemia. A randomized, multicenter, double-blind study comparing the safety and efficacy of intravenous paricalcitol and calcitriol in hemodialysis patients showed that treatment with paricalcitol resulted in a more rapid reduction in parathyroid hormone concentrations while being associated with fewer sustained episodes of hypercalcemia than calcitriol therapy [14]. 

Because of the above-mentioned reasons, the use of selective vitamin D analogues could be beneficial in patients who are at particularly high risk of hypercalcemia due to other comorbidities or medications. For instance, hypercalcemia is common in granulomatous diseases such as sarcoidosis: 10% of patients with sarcoidosis have mild to severe hypercalcemia and up to 50% have hypercalciuria [60]. The intake of lithium, which is used in the therapy of manic-depressive psychoses, or thiazides, an important drug in heart failure therapy, can also lead to hypercalcemia. Hypercalcemia can cause abdominal pain, nausea, and vomiting, as well as cardiac arrhythmias or psychiatric symptoms such as delirium, psychosis, and stupor [61,62,63]. 

## 4. Potential Use of Vitamin D Analogues in Geriatrics

Approximately 90% of the elderly population have impaired vitamin D levels [64]. On the one hand, the decreasing 7-DHC content of the aging skin as well as malnutrition and decreasing gastrointestinal absorption leads to a deficiency of the substrates necessary for vitamin D synthesis. On the other hand, the elderly and chronically ill are constantly housebound due to immobility, so their skin is inadequately exposed to sunlight, which interferes with vitamin D synthesis. In addition, drug interactions or chronic diseases, such as renal or hepatic insufficiency, may inhibit the enzymes required for vitamin D synthesis or the synthesis of DBP, as described before. Therefore, supplementation with vitamin D analogues could have a special place in geriatrics. Some vitamin D analogues do not require the presence of the highly regulated mitochondrial cytochrome P450-like hydroxylases, which may be reduced in old age because of chronic diseases and polypharmacy. However, due to the lack of studies comparing the use of vitamin D analogues with the use of cholecalciferol in geriatric patients, it is explicitly not possible to recommend therapy at the current time.

## 5. Alzheimer’s Disease and Vitamin D Analogues

A common geriatric disease whose prevalence increases with age is dementia. While the average European prevalence rate in the over-65 age group is 8.46%, it is as high as 36.32% in the over-90 age group, meaning that one in three 90-year-olds has dementia. AD is the most common form of dementia, accounting for 50–70% [65]. The World Alzheimer’s Report estimates that there are currently more than 55 million dementia sufferers worldwide, of whom around 1.7 million live in Germany. On a national level, the prevalence is expected to double by 2050, and globally more than three times as many people could suffer from the disease by then [66,67]. AD is thus gaining increasing social and economic importance. 

Vitamin D deficiency is reported to double the risk of developing dementia, and the risk of developing AD can increases by 20% [68,69,70,71,72]. In addition, meta-analyses and longitudinal cohort studies show an association between low vitamin D levels and cognitive decline, which is considered a typical clinical symptom of AD [71,73,74,75,76,77]. Patients with AD also had significantly lower levels of vitamin D in serum and cerebrospinal fluid than the general population in different studies [78,79,80,81,82]. Studies investigating whether compensating for a vitamin D deficit protects against AD or whether the symptomatology and pathology can be improved by vitamin D supplementation have produced controversial results. Some studies reported slower progression of symptomatology in AD or altered plasma Aβ_1–40_ levels after vitamin D supplementation, which would suggest a reduced risk of AD [83,84,85] (see Table 3). The time to onset of psychotic symptoms in patients with AD was also significantly prolonged by vitamin D supplementation. In the study this was attributed to altered AD and psychosis-related genes after vitamin D supplementation, among other factors [86]. Vitamin D could thus provide an interesting additional therapy for neuropsychiatric symptoms in patients with AD, which is particularly relevant since antipsychotic drugs are suspected of increasing the risk of death in patients with AD [87]. However, there are also studies reporting no significant association between vitamin D levels, AD, and cognitive function, with vitamin D supplementation providing no benefit [88,89]. The inhomogeneous study situation might be partly due to methodological/logical reasons such as a high variability in dosage and dosage form of the administered vitamin D preparations or a small number of participants and short observation periods. In addition, it is remarkable that to our knowledge no larger study has so far tested vitamin D analogues in patients with AD. Only one study in hemodialysis-dependent patients with secondary hyperparathyroidism measured the effects of maxacalcitol on the Dementia Assessment Sheet for Community-based Integrated Care System. After the study participants received the vitamin D analogue maxacalcitol intravenously three times a week for 12 months, there was no relevant change in the dementia test. However, the study specifically aimed to influence calcification with maxacalcitol, and study participants achieved almost complete performance in cognition and activity at baseline, which may explain why no relevant improvement was observed during the selected time [90]. Additionally, from a mechanistic perspective, it can be assumed that vitamin D and its analogues might have an impact on AD, since important characteristic neuropathological and neurochemical features of AD are associated with vitamin D. In the following paragraph, the preventive and therapeutic potential of a treatment of vitamin D analogues in relation to AD will be discussed in more detail.

### 5.1. Vitamin Analogues, Extracellular Amyloid Plaques, and Intraneuronal Neurofibrillary Tangles

AD is characterized by intracellular and extracellular protein deposits in brain tissues which, among other things, disrupts nutrient transport and neuronal communication. In addition, the supply of oxygen and energy is impeded by protein deposits in vessels. In the longer term, this leads to degeneration of neurons, cell death, and brain atrophy [91]. The hippocampus and cortical brain regions are particularly affected, resulting macroscopically in enlargement of the sulci and ventricles [92]. In parallel, inflammatory processes occur in the context of chronic inflammation, as the microglia aim to remove the accumulated proteins. In addition to its central role in bone formation, vitamin D also performs important functions in the brain. It regulates the brain’s own inflammatory reactions, supports neuronal proliferation, and thus contributes overall to neuroprotection. Neurotransmitter levels, neurotrophic factors, and calcium balance in the brain are also regulated by vitamin D [93,94,95]. In the hippocampus of rats treated with calcitriol, neuronal density increased and brain atrophy decreased, and incubating cortical and hippocampal neurons with calcitriol decreased glutamate-induced neurotoxicity. Overall, it can be assumed that most vitamin D analogues can also have cerebral effects, since, for example, the expression of enzymes necessary for their activation, such as CYP27A1, as well as the vitamin D receptor, are expressed in the brain, and vitamin D metabolites have been shown to cross the blood–brain barrier [73,74].

The extracellular protein deposits in AD are predominantly composed of aggregated amyloid, which is formed by proteolytic cleavage from amyloid precursor protein (APP) [96]. Reduction of cerebral Aβ levels is being pursued as a potential disease-modifying therapeutic approach. In 2021, antibody therapy for AD was approved in the United States with the monoclonal antibody aducanumab, which targets soluble and aggregated Aβ. However, the approval has been controversial and further confirmatory studies through 2030 will assess the clinical benefit [97]. The European Medicines Agency rejected a marketing authorization application for aducanumab in 2021 due to a lack of evidence of efficacy and potential serious side effects, such as brain swelling and bleeding. 

Vitamin D and vitamin D analogues have been reported to lower Aβ levels in preclinical studies and vitamin D tolerability has been confirmed in several clinical studies. These preclinical studies include cell culture studies as well as animal studies. In a previous study, it was shown that the therapeutically used analogues maxacalcitol, calcipotriol, alfacalcidol, paricalcitol, and doxercalciferol reduced Aβ levels in the human neuroblastoma SH-SY5Y cells stably transfected with human APP^695^, the major isoform in neurons (see Table 3). There were no significant differences in effect size between the individual analogues or between the analogues and calcifediol [98]. Follow-up studies confirmed these results for some analogues in vivo. APP transgenic mice injected intraperitoneally with 200 ng/kg paricalcitol every other day for 15 weeks had significantly fewer Aβ oligomers. Moreover, the vitamin D analogue maxacalcitol was tested in rats in which pathology of AD had previously been simulated by injection of lipopolysaccharides. The rats were injected intraperitoneally with 1 µg maxacalcitol per kilogram body weight twice daily. Subsequently, enzyme-linked-immunosorbent-assay (ELISA) was used to determine the Aβ concentration in brain tissue. The Aβ concentration of the lipopolysaccharide-treated rats was significantly higher than that of the control group and could be halved again by an addition 4-week injection of maxacalcitol. The cognitive performance of maxacalcitol-treated rats was also significantly improved [99,100].

**Table 3:** Summary of the main results observed in cell-based studies, animal models, or clinical trials regarding vitamin D and its analogues and AD.

Author	Year	Type of Study/Duration/*n*	Main Findings
Shen, L. et al. [68]	2015	Meta-Analysis/until February 2015/2 prospective cohort studies and 3 cross-sectional studies (10,019 participants)	Vitamin D deficiency (25(OH)D level < 50 nmol/L) was associated with a 21%increased risk of developing AD compared to adequate vitamin D levels (25(OH)D level > 50 nmol/L).
Jayedi, A. et al. [69]	2019	Meta-Analysis/until September 2017/7 prospective cohort studies and 1 retrospective cohort study (28,354 participants)	The risk of developing AD decreased with increasing vitamin D levels up to ∼35 ng/mL. Vitamin D insufficiency (10–20 ng/mL) resulted in HR of 1.19 (95% CI: 0.96, 1.41) and vitamin D deficiency (<10 ng/mL) resulted in HR of 1.31 (95% CI: 0.98, 1.65).
Chai, B. et al. [70]	2019	Meta-Analysis/until 1 January 2019/12 prospective cohort studies and 4 cross-sectional studies (21,784 participants)	Vitamin D deficiency (<20 ng/mL) was significantly positively associated with the risk of dementia and AD. In the case of vitamin D deficiency (<20 ng/mL) the pooled HR was 1.34 (95% CI: 1.13, 1.60) in comparison to sufficient vitamin D supply.
Jia, J. et al. [84]	2019	RCT/12 months/210 participants with AD	Intervention with 800 IU/day of vitamin D in patients with AD may ameliorate the cognitive function and reduce Aβ-associated biomarkers. The results of the intervention group showed significant amelioration of plasma Aβ42, APP, BACE1, APP mRNA, BACE1 mRNA (*p* < 0.001) levels and information, arithmetic, digit span, vocabulary, block design and picture arrange scores (*p* < 0.05) unlike the control group.
Miller, B. et al. [83]	2016	RCT/8 weeks/24 participants	Intervention with vitamin D (50,000 IU/week) resulted in greater plasma Aβ40 change than in the control group (+14.9 ± 12.0 and +12.8 ± 12.8 pg/mL; *p* = 0.045; effect size, 0.228) especially in older participants (≥60 y), where the change in Aβ40 was + 18.3 ± 33.6 and −3.2 ± 44.5 pg/mL for vitamin (*n* = 4) and placebo (*n* = 4) groups (effect size, 0.295), which insinuates reduced brain Aβ.
Cellular and Animal Studies			
Saad El-Din, S. et al. [100]	2020	In vivo study/lipopolysaccharide-induced rat model of AD/maxacalcitol by intraperitoneal injection in a dose of 1 μg/kg/day, twice a day for 4 weeks	Improvement of cognitive dysfunction; increased expression of Nrf2; decreased neuro-inflammation/amyloid-β load/hyperphosphorylation of MAPK-38, ERK1/2, tau proteins.
Fan, Y. et al. [99]	2019	In vivo study/APP/PS1 transgenic mice/paricalcitol by intraperitoneal injection in a dose of 200 ng/kg every two days for 15 weeks	Reduction of Aβ formation by acceleration of lysosomal BACE1 degradation, inhibition of neuronal loss.
Grimm, M. et al. [98]	2017	In vitro and ex vivo study/neuroblastoma cells or vitamin D-deficient mouse brains/incubation of 100 nm calcifediol or maxacalcitol/calcipotriol/alfacalcidol/paricalcitol/doxercalciferol	Significantly decreased Aβ production and increased Aβ degradation, mediated by affecting the activity, protein level, and expression of β- and γ-secretases.

Aβ is generated from the cleavage of APP by β-secretase and the subsequent processing by γ-secretase and can then be degraded or transported by different mechanisms. APP can also be processed by α-secretase in a non-amyloidogenic processing pathway preventing Aβ release. In patients with AD, an imbalance occurs due to increased amyloidogenic APP processing and/or a simultaneously reduced Aβ elimination, resulting in increased Aβ levels [101]. It has been shown that vitamin D analogues reduce amyloidogenic APP processing on the one hand by inhibiting β- and γ-secretases via direct and indirect mechanisms. On the other hand, vitamin D analogues can enhance Aβ degradation by increasing expression and activity of the major Aβ degrading enzyme, the zinc metalloendopeptidase neprilysin [98]. In addition, vitamin D analogues enhance α-secretase activity, which initiates non-amyloidogenic processing of APP.

Maxacalcitol has also been studied for its ability to reduce hyperphosphorylated tau proteins. These proteins form the major component of intracellular protein deposits. Maxacalcitol was able to significantly decrease hyperphosphorylation of tau in rat brain tissue. This supports the hypothesis that vitamin D analogues may affect the pathology of AD via pleiotropic mechanisms, thus providing a potential causal therapeutic approach [100]. 

### 5.2. Vitamin D, Its Analogues, and Parallels to Antidementia Drugs

At present, there are no drugs approved in Europe for the treatment of AD that are able to causally treat the pathology of AD. To date, only a symptomatic therapy is recommended, with drugs that affect the neurotransmitter levels that have become imbalanced. Decreased acetylcholine levels have been described in patients with AD, which is associated with learning and memory disorders. Accordingly, anticholinergics, which inhibit the action of acetylcholine, are suspected of increasing the risk of dementia [102]. Dopamine levels are also decreased in patients with AD and dysfunction of the dopaminergic system correlates with apathy, a common behavioral symptom in patients with AD [103,104,105]. Furthermore, patients with AD exhibit increased glutamate release, which stimulates the N-methyl-D-aspartic-acid (NMDA) receptor, resulting in increased intracellular calcium levels that can lead to cell apoptosis. Thus, the use of acetylcholinesterase inhibitors such as donepezil, galantamine, or rivastigmine raises the decreased acetylcholine level, while NMDA antagonists such as memantine affect the increased glutamate release. This provides short-term symptomatic improvement in the dementia stage but is not able to permanently stop the progression of the disease. In addition, the therapy often has numerous side effects resulting in discontinuation of acetylcholinesterase inhibitor therapy after an average of 14 months [106,107,108]. Moreover, according to a meta-analysis, only 9% of patients responded to treatment with acetylcholinesterase inhibitors and showed an effect beyond the placebo effect [109]. 

**Figure 4:**
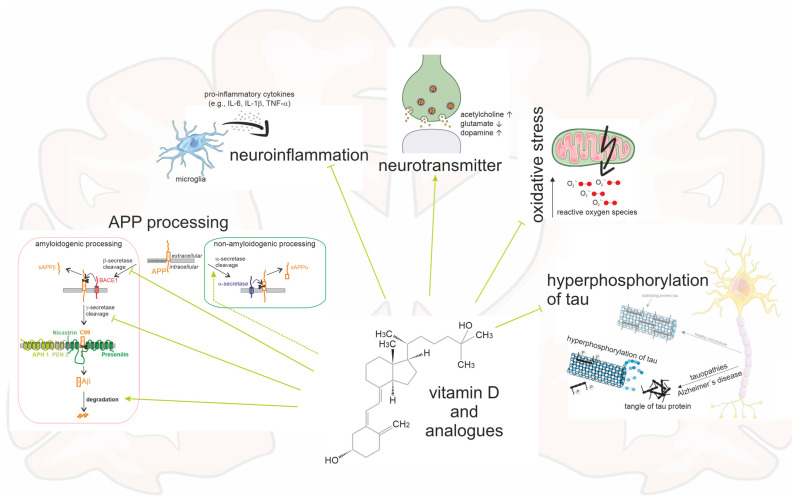
Schematic of an AD brain and the multiple impacts caused by vitamin D and its analogues. Vitamin D and its analogues have an inhibiting influence on Aβ plaques by reducing the amyloidogenic processing and promoting non-amyloidogenic processing and Aβ degradation. Moreover, neurofibrillary tangles, oxidative stress, and neuroinflammation are diminished. The neurotransmitters acetylcholine and dopamine are enhanced, and glutamate is degraded.

Vitamin D and its analogues have been shown to regulate these important neurotransmitter levels as well. They could act in a similar way to the antidementia drugs, but in contrast to the antidementia drugs, they have few side effects. However, the observed effect strength suggests that vitamin D should be used in combination with other drugs. Rats treated with vitamin D showed significantly increased activity of choline acetyl transferase in some brain regions; this enzyme catalyzes the synthesis of acetylcholine and a decrease in its activity correlates with the severity of dementia [110,111]. Furthermore, in an AD model in rats, in which dementia was simulated by streptozotocin injection, vitamin D supplementation normalized pathologically elevated acetylcholinesterase, which degrades acetylcholine [112]. Vitamin D promoted differentiation of dopaminergic neurons and calcitriol supplementation in mice and rats significantly increased dopamine levels [113,114,115]. Conversely, a vitamin D deficit is associated with a dopaminergic deficit because, for example, the expression of catechol-O-methyltransferase, which is necessary for the synthesis of dopamine, is decreased [116]. Furthermore, vitamin D may reduce glutamate-induced neurotoxicity in a manner similar to the NMDA receptor antagonist memantine [117,118]. A study in patients with AD examined the combination of vitamin D and memantine in terms of cognitive improvement. For this purpose, the mini mental state examination (MMSE) was collected before and after 6 months of taking vitamin D, memantine, or both. While taking vitamin D or memantine alone did not significantly affect the MMSE scores, the combination of both preparations showed an improvement in MMSE scores by an average of four points [119,120] further emphasizing the beneficial effects of vitamin D in combination with other drugs. In cell culture, the effect of both vitamin D and memantine on Aβ- or glutamate-induced axon toxicity was tested. The combination of vitamin D and memantine was shown to be protective, whereas pure memantine or vitamin D showed little or no effects [121]. Thus, there is evidence for a synergistic and potentiating mode of action for vitamin D and memantine. High glutamate levels and activated NMDA receptors decrease the expression of renal 1α-hydroxylase [122]. If this is transferable to extrarenal cerebral 1α-hydroxylase, the 1α-hydroxylated vitamin D analogues may act more potently than vitamin D, because they act without the potentially downregulated 1α-hydroxylase. For patients with advanced liver failure, the use of memantine is contraindicated, and insufficient data are available on some hepatically eliminated cholinesterase inhibitors, such as donepezil. Here, 25-hydroxylated vitamin D analogues, which act independently of 25-hydroxylases, may gain relevance. Figure 4 summarizes the multiple ways in which vitamin D analogues interact with the pathology of AD.

### 5.3. Vitamin D Analogues and Non-Pharmacological Approaches for Alzheimer’s Disease

As a non-drug treatment modality, exercise therapy in particular shows benefit for cognitive function in patients with AD. Regular physical activity slows memory loss in patients with AD [123,124] and increases cerebral blood flow [125,126]. In a cohort study conducted on patients at risk for AD, physical activity significantly correlated with Aβ load, glucose metabolism, and hippocampal volume [127]. Vitamin D supplementation could maintain mobility and thereby access to exercise therapies for a longer period. It is known that vitamin D supplementation significantly reduces fracture and fall risk and maintains musculoskeletal functionality [61,128,129,130,131]. Reducing fracture risk can prevent hospitalization and surgery. This is also of high relevance, because some types of anesthesia led to an increase of Aβ load in neurons in vitro, suggesting that certain anesthetics accelerate the appearance of symptoms in AD [132]. The vitamin D analogues, especially alfacalcidol, might be particularly interesting in this context, as one study showed that alfacalcidol was more effective in protecting against spinal fractures than calcitriol [133]. 

Maintaining mobility and promoting exercise also helps reduce the cardiovascular risk profile. Most cardiovascular risk factors, including diabetes mellitus, arterial hypertension, hypercholesterolemia, smoking, obesity, and physical inactivity, are associated with increased risk of AD and vitamin D deficit may lead to an increased incidence of cardiovascular disease [6,134,135,136,137,138]. Minimizing the risk profile is of great importance in the prevention of AD, and vitamin D analogues may contribute to this too. The vitamin D analogue paricalcitol was shown in a meta-analysis to significantly reduce the risk of cardiovascular events in renal failure patients and to maintain reduced arterial flexibility in the setting of progressive atherosclerosis [139,140]. A preclinical study in mice compared the effect of vitamin D3 and different vitamin D analogues, including calcifediol, alfacalcidol, and doxercalciferol, on cholesterol levels in mice fed a high-cholesterol Western diet. Except for doxercalciferol, all vitamin D analogues studied lowered cholesterol levels. Alfacalcidol showed stronger effects than calcifediol and calcitriol [141]. Cholesterol-lowering interventions, such as the regular use of statins, have been shown to significantly reduce the risk of developing dementia in some studies. Vitamin D analogues could therefore also represent a potential preventive therapeutic by influencing lipid homeostasis [142]. 

Furthermore, vitamin D analogues also influence blood pressure via interactions with the blood pressure regulating renin–angiotensin–aldosterone system, among others [143]. For example, eight weeks of paricalcitol treatment significantly reduced myocardial expression of renin and angiotensinogen in rats, and doxercalciferol reduced expression of renin and the angiotensin II receptor in mice [144,145]. Treatment with alfacalcidol reduced systolic blood pressure by 6 mmHg and diastolic blood pressure by 5.8 mmHg in a clinical trial [146,147]. However, other vitamin D analogues, e.g., paricalcitol, did not show significant antihypertensive effects [148]. Improved brain perfusion, for example, could positively affect the permeability of the blood–brain barrier, leading to better Aβ clearance. Antihypertensive therapies, such as angiotensin-converting enzyme inhibitors or angiotensin receptor antagonists, have already been associated with decreased Aβ concentrations in the brain, but according to a meta-analysis, cognitive abilities did not improve significantly [141,144,145,149]. Since, as shown in Table 2, some antihypertensives interact with the enzymes necessary for vitamin D synthesis, the parallel use of vitamin D analogues that act independently of 25-hydroxylase could be of particular relevance.

### 5.4. Vitamin D Analogues and Benefits in Comorbidities of Alzheimer’s Disease

Overall, it can be noted that most patients with AD are over 65 years of age and are often multimorbid, meaning that they have at least two diseases and that their polypharmacy can lead to confusing drug interactions [150]. Diseases such as renal insufficiency, in which 1α-hydroxylase is decreased, are a common comorbidity of AD because they have the same risk factors as arterial hypertension and diabetes mellitus, and the increased uremic toxins may promote dementia. The use of hydroxylated vitamin D analogues could hypothetically be beneficial here because of the altered pharmacological properties.

Because patients with AD are at increased risk for developing depression or epilepsy, antidepressants or antiepileptic drugs are also often used in these patients [151]. However, these can interact with the enzymes needed to synthesize vitamin D. A study of patients with depressive symptoms showed that six weeks of ergocalciferol supplementation significantly improved mental health status and significantly decreased depression and anxiety. Participants not taking an antidepressant in parallel responded better to supplementation, which may have been due to the limited metabolism of ergocalciferol by parallel antidepressant use [152]. Vitamin D analogues, which work independently of CYP enzymes, might be more suitable for combination therapy in this case. The same applies to parallel treatment with antiepileptics that affect vitamin D synthesis. Epilepsy and AD favor each other, making epilepsy a common comorbidity in patients with AD. Aβ and tau protein on the one hand increase neuronal excitability, leading to increased seizures, and on the other hand, Aβ deposits increase after an epileptic seizure. Paricalcitol has been shown to have anticonvulsant properties in animal studies, making it particularly useful for patients with AD and epilepsy [153].

In conclusion, vitamin D derivatives, including calcitriol, alfacalcidol, and paricalcitol, have shown potential advantages in the treatment of various metabolic and degenerative diseases under special conditions. These advantages include improved efficacy and reduced dosing requirements compared to cholecalciferol. However, the administration of analogues warrants prudent consideration due to their potential risk for adverse effects such as hypercalcemia, hyperphosphatemia, and hypercalciuria. It is also important to note that vitamin D supplementation should not be used as a standard and carelessly given substitute for established treatments and should be tailored to the individual patient’s needs. More studies are needed to fully understand the advantages and limitations of these derivatives, and to establish safe and effective dosing recommendations.

## Figures and Tables

**Table 2:** Summary of different inhibitors and inducers that affect the enzymes of the cytochrome P450 superfamily.

Enzyme and Effect	Drugs
partial CYP27A1 inhibitors [36]	anti-hypertensive drugs ○ calcium channel blockers (clevidipine, felodipine, nicardipine, nilvadipine, nimodipine) ○ angiotensin II receptor antagonist candesartan (but not losartan, irbesartan, eprosartan, telmisartan) anti-HIV medication ○ non-nucleoside reverse transcriptase inhibitors (delavirdine and etravirine) anti-cancer drugs ○ abiraterone, dasatinib, nilotinib, and regorafenib
CYP3A4 inhibitors [38]	macrolide antibiotics ○ erythromycin, clarithromycin anti-cancer drug ○ tamoxifen anti-depressants ○ fluoxetine, midazolam anti-hypertensives ○ verapamil, dihydralazine anti-HIV medication ○ indinavir, nelfinavir, ritonavir
CYP3A4 inducers [39]	anti-epileptic drugs ○ phenobarbital, phenytoin, valproic acid anti-microbial drug ○ rifampin

## Data Availability

Not applicable.
